# Ecological and evolutionary implications of allometric growth in stomach size of brachyuran crabs

**DOI:** 10.1371/journal.pone.0207416

**Published:** 2018-11-09

**Authors:** Blaine D. Griffen, Zachary J. Cannizzo, Mustafa R. Gül

**Affiliations:** 1 Department of Biology, Brigham Young University, Provo, UT, United States of America; 2 Marine Science Program, School of the Earth, Ocean, and Environment, University of South Carolina, Columbia, SC, United States of America; Universidad de Cádiz, Facultad de Ciencias del Mar y Ambientales, SPAIN

## Abstract

Individual characteristics often scale allometrically with organismal body size and the form of this scaling can be influenced by ecological and evolutionary factors. Examining the specific form of this scaling can therefore yield important insights into organismal ecology and evolution and the ability of organisms to respond to future environmental changes. We examine the intraspecific allometric scaling of stomach volume with body mass for 17 species of brachyuran crabs. We also examine how this scaling is influenced by dietary strategy, maximum body size, and activity level, all while controlling for phylogenetic relationships between the species. We show that the slope and intercept of the allometric scaling relationships vary across species and are influenced by all three ecological factors examined here, as well as by evolutionary relationships. These results highlight potential divergent strategies in stomach growth taken by different groups of crabs and highlight potential limitations that may be imposed on the ability of this group of organisms to respond to warming trends expected with climate change.

## Introduction

The study of scaling in individual characteristics with body size, or allometry, has been a constant theme of ecology, anatomy, morphology, physiology, developmental biology, and evolutionary biology for nearly a century [1]. Originally focusing on relative scaling of metabolism [2] and anatomical and physiological traits [3], the focus has more recently broadened to encompass scaling relationships for a wide range of ecological factors, including the acquisition and use of resources [4], prey selection [5], reproductive performance [6], population abundance [7], and community and ecosystem properties [8]. Given the importance of allometric relationships, the form of this scaling has received a considerable amount of attention and has sparked spirited debate (e.g., [9–12]). (For recent reviews of developments in metabolic scaling, see [13] and [14].) Disagreements have focused on the appropriate assumption for the scaling exponent (*b*) in relating metabolic functions with body mass (*M*) in the equation:

$$Metabolic\;function=aM^{b}$$ (Eq 1)

Eq 1 can be linearized by expressing it on a log-log scale. In this case, the exponent (*b*) becomes the slope, and the scaling factor (*a*) becomes the intercept in the linear relationship. We therefore hereafter refer to these as the slope and intercept.

Much of the debate surrounding scaling of metabolic processes and structures that support metabolism has focused on theoretically predicted values for the slope of the allometric relationship [3]. However, there is often considerable variation and deviation from theoretical expectations across organisms, and much of the controversy likely arises from the focus on this mean theoretical value, while ignoring the variation that can be seen within and between phylogenetic groups [15]. While statistical models can account for this structured variation statistically [15], a larger need is to understand the reason for this variation ecologically.

Additionally, while much of the focus in allometric scaling has centered on the slope (i.e., the 0.75 power rule), recent developments in the scaling of metabolism demonstrate that the intercept in the allometric relationship is also important, that the slope and the intercept interact, and that both can be influenced by ecological drivers [13,16–18]. For instance, the metabolic level boundaries hypothesis predicts that the slope of the allometric relationship should covary with the intercept, and that the correlation between these two parameters can be negative or positive, depending on the metabolic demands of the organisms, and whether metabolic rates are resting (negative covariation) or active (positive covariation) [13,16]. A wide range of organisms, from fish [19] to mammals and birds [20] seem to support this hypothesis.

In addition to metabolic rates, the allometric scaling of numerous morphological features and physiological functions with body mass have been examined. And indeed, for metabolism to scale with body mass, theory predicts that the biological machinery responsible for acquiring and processing the resources that fuel metabolism must also scale with body size [13,21–23]. Empirical evidence seems to support this expectation, as the size of the digestive tract (and other body systems) generally increases with body mass across species of mammals [24], insects [25], fish [26], etc.

As with allometric scaling of metabolic rates, we may expect that both the slope and the intercept in allometric scaling of stomach size should be important and that both may be influenced by ecological factors. Previous work on allometric scaling of the gut has focused exclusively on the slope of the relationship between gut size and body mass. It is anticipated that gut volume will scale linearly (i.e., isometrically with a slope ~1) with body mass [27], and empirical evidence seems to support this assumption [28, 29].

Several ecological factors influence energy demands and may therefore be important for allometric scaling of stomach size across ontogeny. For example, carnivores and omnivores are often more mobile than herbivores, requiring larger home ranges to meet their energetic and nutritional needs [30], and consequently will often have higher basal metabolic rates [31], though in general this will depend on the digestibility and nutritional value of the diet [32]. Additionally, species show a wide range of activity levels, from those that are sessile and essentially immobile, to those that are highly mobile and active, with metabolic demand being higher for more active species [33, 34]. Finally, there is also considerable variation in the maximum body size attainable across species, with overall metabolic demand being greater for species with larger body sizes. Although diet, activity level, and body size certainly vary across highly disparate species, there is considerable variation in these three ecological traits even within phylogenetic groups.

Here we examine the intraspecific allometric scaling of stomach volume with body mass across 17 species of marine brachyuran crabs. As with other consumers, the stomach size of crabs is strongly influenced by diet strategy, with more herbivorous species having larger stomachs than their carnivorous counterparts [35]. In addition, males and females often have very different energetic costs associated with reproduction (e.g., [36]), leading to differences in diet and food consumption strategies (e.g., [37]). The same can also be said for crabs across different habitat types [38]. Given the strong impact of diet strategy on stomach volume, and the interaction between dietary intake and gender or habitat, we may expect that these factors will also influence the scaling of stomach volume with body size. We therefore tested the following three hypotheses. 1) Stomach volume will scale approximately isometrically with body mass, with some variation in this scaling between species and between males and females and also between habitats. 2) The slope of the allometric relationship of stomach volume with body mass will increase with the amount of herbivory in the diet, activity level, and maximum body size of the species examined. 3) The intercept of the allometric relationship of stomach volume with body mass will also increase with diet, activity level, and maximum body size of the species examined.

## Methods

Griffen and Mosblack [35] examined 15 different crab species to determine whether stomach size could be used to infer diet and consumption rates. For each species, they dissected numerous crabs (n = 35–126) and measured the width of the stomach for each. Though not reported in Griffen and Mosblack [35], they also measured the dry mass for each crab. We used these same data, together with data on two additional species, the mangrove tree crab *Aratus pisonii* (n = 385) and the Atlantic ghost crab *Ocypode quadrata* (n = 146), to examine allometric changes in stomach volume with body mass.

For a full description of the dissection procedures, see Griffen and Mosblack [35]. Briefly, crabs were collected in June and July 2009 from the locations given in Table 1. Upon collection, crabs were immediately euthanized by placing them in ice water. Upon returning them to the laboratory, each crab was dissected by removing the dorsal carapace and stomach with forceps. The width of the cardiac stomach was then measured to the nearest 0.1 mm on the anterior ventral edge using a dissecting microscope. The dry mass of each crab was determined by drying to constant weight at 70°C.

**Table 1:** Species examined (column 1), sample size (column 2), GPS coordinates of sampling location (column 3), mean Slope (and standard error) of the allometric log-log relationship where M is for male and F is for female crabs (column 4), mean Intercept (and standard error) of the log-log allometric relationship where M is for male and F is for female crabs (column 5), Genbank accession numbers for 16S rRNA data used in constructing the phylogenetic tree (column 6). Only a single slope and intercept are given when the allometric relationship did not differ between males and females (α = 0.05). The slope and intercept for each of the relationships given is highly significant (*P* < 0.0001).

Species	Sample size	Sampling location(s)	Slope (S.E.)	Intercept (S.E.)	Genbank accession numbers
*Aratus pisonii*	380	Mangrove:			HG939507.1
		27^o^33’33.66”N	M: 0.839 (0.032)	M: 3.941 (0.022)	
		80^o^19’53.32”W	F: 0.758 (0.048)	F: 3.997 (0.036)	
		Marsh:			
		30^o^0’49.18”N	M:0.701 (0.076)	M: 3.659 (0.097)	
		81^o^20’42.54”W	F:0.894 (0.083)	F: 3.967 (0.118)	
		Dock:			
		30^o^7’57.52”N	0.710 (0.049)	3.753 (0.026)	
		81^o^23’8.03”W			
*Armases cinereum*	70	33°20’5.09”N	1.035 (0.028)	4.267 (0.025)	AJ784010
		79°11’37.51”W			
*Callinectes sapidus*	66	33°21’1.93”N	1.185 (0.040)	4.285 (0.085)	AJ298190
		79°11’27.67”W			
*Cancer irroratus*	74	43°9’56.17”N	1.062 (0.035)	3.502 (0.044)	AJ130812
		70°35’30.97”W			
*Carcinus maenas*	125	43°2’15.07”N	M: 1.262 (0.078)	M: 2.280 (0.157)	AY583901
		70°42’51.97”W	F: 1.091 (0.043)	F: 3.521 (0.048)	
*Dyspanopeus sayi*	63	43°5’25.31”N	0.791 (0.37)	2.964 (0.044)	AJ274694
		70°51’52.29”W			
*Eurypanopeus depressus*	57	33°20’58.17”N	0.849 (0.050)	3.577 (0.093)	KT959502.1
		79°11’19.55”W			
*Eurytium limosum*	54	33°20’5.38”N	0.845 (0.036)	2.829 (0.047)	AJ274696
		79°11’38.43”W			
*Hemigrapsus sanguineus*	124	43°2’13.27”N	M: 1.074 (0.015)	M: 2.577 (0.032)	EU367395
		70°42’56.02”W	F: 1.168 (0.025)	F: 2.601 (0.037)	
*Ocypode quadrata*	146	Pristine:	1.369 (0.084)	2.944 (0.174)	KU313182.1
		33°11’8.33”N			
		79°11’9.72”W			
		Medium Impact:	1.515 (0.133)	2.701 (0.229)	
		33°28’13.152”N			
		79° 6’59.37”W			
		High Impact:	1.179 (0.148)	3.528 (0.204)	
		33°36’18.95”N			
		78°58’19.44”W			
		High+Vehicle:	1.209 (0.145)	4.188 (0.167)	
		33°44’8.28”N			
		78°49’27.40”W			
*Panopeus herbstii*	123	33°20’58.17”N	M: 0.728 (0.032)	M: 3.460 (0.046)	AJ130815
		79°11’19.55”W	F: 0.708 (0.060)	F: 3.584 (0.052)	
*Panopeus obesus*	35	33°21’4.38”N	M: 0.602 (0.032)	M: 3.375 (0.067)	AJ274680
		79°11’29.00”W	F: 0.772 (0.061)	F: 3.130 (0.095)	
*Rhithropanopeus harrisii*	76	43°5’25.31”N	M: 0.989 (0.063)	M: 2.827 (0.119)	AJ274697
		70°51’52.29”W	F: 1.031 (0.096)	F: 3.064 (0.222)	
*Sesarma reticulatum*	81	41°55’34.80”N	0.932 (0.011)	4.179 (0.014)	AJ225867
		70°3’30.94”W			
*Uca minax*	81	33°20’5.82”N	0.805 (0.029)	4.056 (0.020)	Z79670
		79°12’18.48”W			
*Uca pugilator*	105	33°19’42.70”N	M: 0.659 (0.027)	M: 3.582 (0.022)	Z79659
		79°12’30.64”W	F: 0.621 (0.031)	F: 3.631 (0.033)	
*Uca pugnax*	69	33°19’55.97”N	0.852 (0.018)	3.850 (0.044)	Z79675
		79°11’56.32”W			

The exception to the procedures described above were data from *A*. *pisonii* and *O*. *quadrata*. *A*. *pisonii* were collected as part of a larger study to examine the physiological responses of this species to a climate-induced range expansion from mangroves into saltmarshes [38, 39]. Samples of this species were collected at various times from saltmarsh, dock piling, and mangrove habitats during the summers (June-September) of 2015 and 2016. *O*. *quadrata* were collected as part of a study to examine the influence of human disturbance of sandy beaches throughout South Carolina, USA. Samples of this species were collected in summer months of 2016 from beaches that were either pristine, moderately impacted by people, heavily impacted by people, or heavily impacted by both people and vehicles [40]. Following collection, crabs were processed as described above for the other species.

Given that the shape of the crab cardiac stomach closely resembles that of a triangular pyramid, we determined the stomach volume for each crab using the measured stomach width and using the equation of a triangle pyramid:

$$Stomach\;volume=\frac{c\sqrt{2}}{12}\times {\mathrm{stomach}\;\mathrm{width}}^{3},$$ (Eq 2)

The value of the parameter, *c*, in this equation was empirically determined to be 0.92 using water displaced by stomachs submerged in a graduated cylinder (R^2^ = 0.987, see Griffen & Mosblack [35]).

We examined the allometric relationship between stomach volume and dry mass for each species separately, and identically, as follows. We first removed outliers, defined as any data points whose studentized residuals fell outside the range of -2.5 to 2.5 [41] (totaling 34 of the 1,710 data points). Organismal growth is generally a multiplicative process [42], and allometry of multiplicative processes is most accurately assessed using linear regressions on log-transformed data [43]. We therefore then used an ANCOVA to examine log stomach volume as a function of log dry weight (continuous variable) and gender (categorical factor). We chose to use the natural logarithm in this study. If the interaction term in this ANCOVA was significant, we conducted individual regressions to examine log stomach volume as a function of log dry weight for each gender separately. If the interaction term was not significant, it was removed. If the main effect of gender was significant after removing the interaction, separate analyses were conducted for each gender. If the main effect was not significant after the interaction was removed, a regression analysis was conducted on the pooled data from males and females. Analyses with *A*. *pisonii* and *O*. *quadrata* were conducted in an analogous manner, but with habitat included as an additional factor.

Allometric relationships can be similar across groups of related species because of the underlying phylogenetic relationships. This is true for metabolism (e.g., [31, 44]), as well as for a wide range of morphological (e.g., [45, 46]) and life-history characteristics (e.g., [47, 48]). Evolutionary relationships are known to strongly influence allometric relationships in crabs by constraining the shape of the carapace and thus the space inside the carapace that is available for vital organs, and for the storage of energy and reproductive products [6]. Thus, evolutionary history may confound our ability to examine the ecological factors that influence allometric growth of the stomach across species. We therefore controlled for phylogenetic relationship in our analyses. The most common method for controlling for the effects of phylogenetic relationship is with phylogenetically independent contrasts [49, 50]. This method, however, assumes that there is only a single value of the metric of interest at each tip of the phylogenetic tree, whereas we had multiple values for some (male and female for some species and different habitats for *A*. *pisonii* and *O*. *quadrata*). Following de Villemereuil & Nakagawa [51], we therefore instead used a general linear mixed effects model to account for phylogenetic relationship when examining the relationship between ecological factors and the slope and intercept of allometric scaling. Specifically, we analyzed the slope and intercept separately using the following procedure.

We fit a linear mixed effects model to the slope or intercept with diet (mean proportion of diet that was herbivory), maximum body size, and activity level as continuous predictor variables. We used proportional herbivory values given in the literature: *A*. *pisonii* [52], *O*. *quadrata* [53], and all other species [35]. For maximum body size we used the maximum carapace width from the samples for each species reported here. However, we found that maximum body size and percent herbivory were negatively correlated (linear regression, R^2^ = 0.35, *P* = 0.0008). We therefore used the residual maximum body size after accounting for differences in diet (i.e., the residuals from the regression of body size on diet). For activity level, we used the estimated distance moved per day. We estimated this daily movement distance using published-mark recapture studies, site fidelity studies, and personal observations for each species. We then standardized values to range from 0 (sedentary) to 1 (highly mobile). We controlled for phylogenetic relationship by including family, genus, and species as nested random factors in the analysis (based on the most comprehensive published phylogeny for brachyurans [54]). We conducted this analysis using lmer from the lme4 package in R. We fit a full model to the data with all possible interactions, as well as all reduced models. We then used AIC to determine which model provided the best fit to the data and report only the results of the best-fitting model here (ΔAIC for all other models was >2.0). The lmer output generally does not provide p-values because determining the degrees of freedom can be difficult for multilevel regressions. However, the p-values can be approximated using either the normal approximation, which works best for large sample sizes [55], or using the Satterwaite approximation [56]. These methods provided identical p-values here.

Slopes and intercepts of allometric relationships can be auto-correlated for purely mathematical reasons. To ensure that the results here are not an artifact of this possible correlation, we also conducted the analysis described in the preceding paragraph with an estimate of the elevation of the relationship (rather than the intercept) that is independent of the slope. Specifically, we used the mass-specific stomach volume at the midpoint of the log-body mass range (*L*), following previous recommendations [57–58]. All other aspects of the analysis were identical to the methods described above.

In order to visually demonstrate how the allometric slopes and intercepts were influenced by phylogenetic relationship in the analysis described above, we also constructed a phylogenetic tree of the species used here and mapped these metrics onto the tree. For this visual demonstration, we were limited to only a single value for the allometric slope and intercept at each branch tip of the tree. We therefore used the slope and intercept of the log-log relationship for all the data within a species pooled. We used 16S rRNA to determine the phylogenetic relationship (see Table 1 for Genbank accession numbers for each species). We used the spiny lobster *Panulirus argus* as the outgroup (Genbank accession number KT716702.1). We conducted the phylogenetic analysis using MEGA 7.0.26 and followed recommendations by Hall [59]. Specifically, we aligned the 16S rRNA sequences across species using MUSCLE. We then constructed a phylogeny using Maximum Likelihood with a Tamura-Nei model and complete deletion of missing data. We estimated the reliability of our tree by bootstrapping, with 1,000 replicates.

## Results

**Fig 1:**
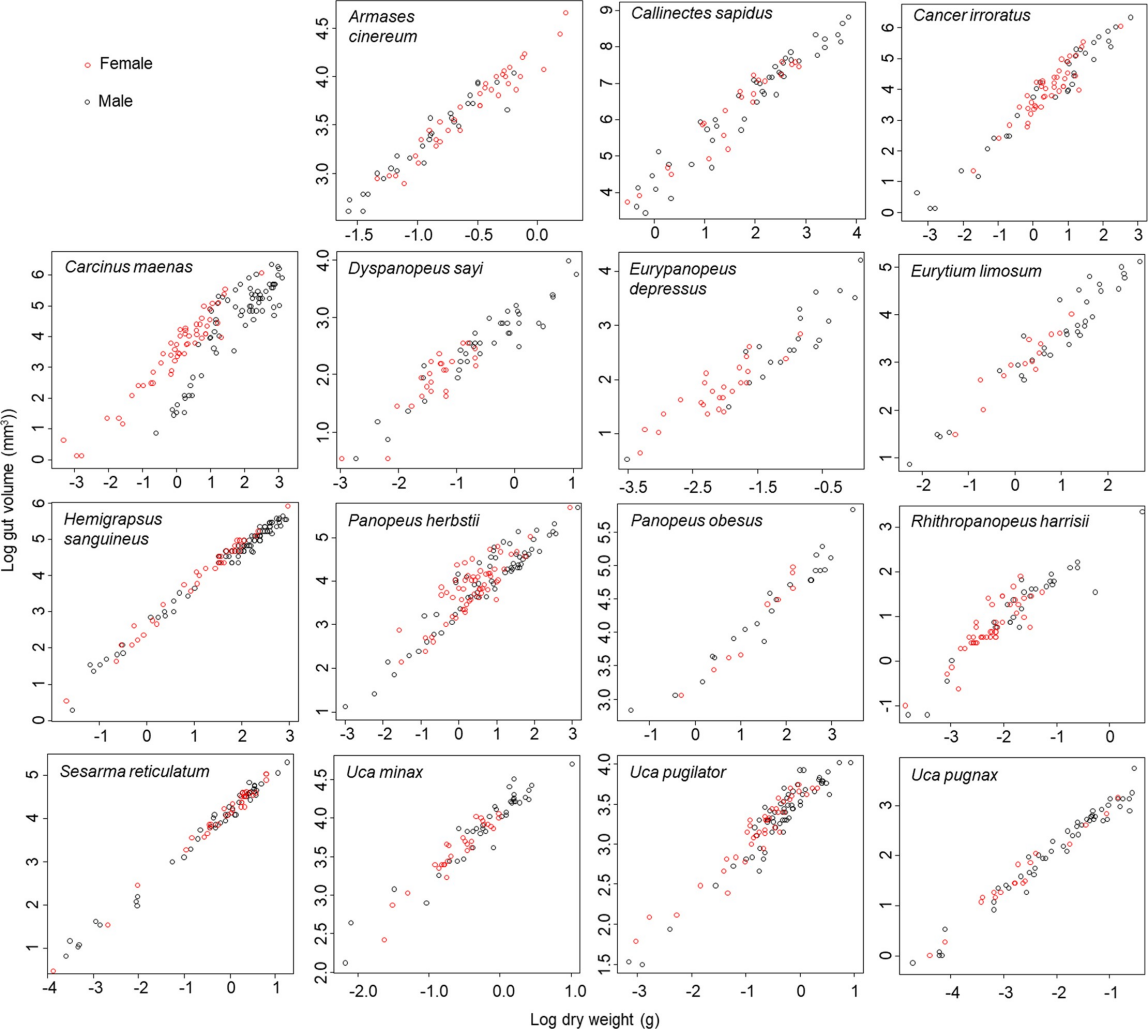
Log stomach volume (mm^3^) as a function of log dry body mass (g) for 15 species of brachyuran crab. Black circles are male and red are female.

**Fig 2:**
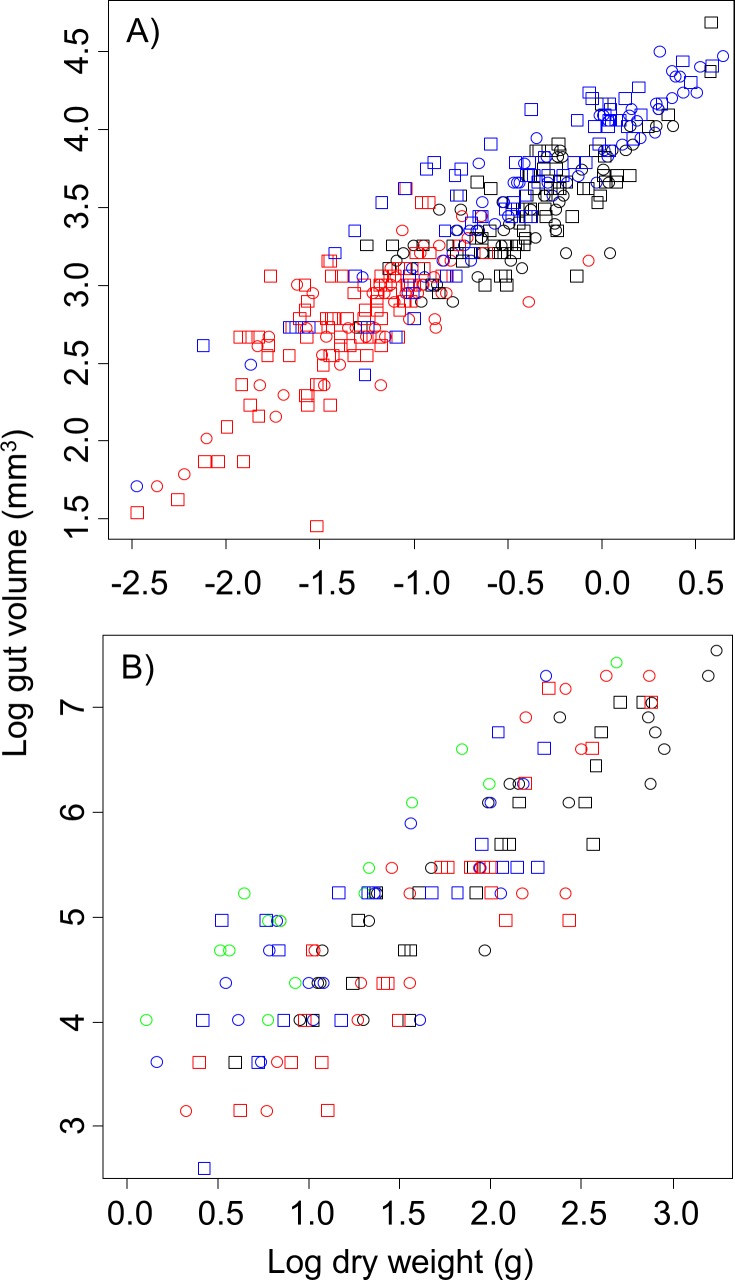
**Log stomach volume (mm**^**3**^**) as a function of log dry body mass (g) for *A*. *pisonii* (A), and *O*. *quadrata* (B).** For both, circles are males, squares are females. For *A*. *pisonii*, black are from a dock habitat, red are from a saltmarsh habitat, and blue are from a mangrove habitat. For *O*. *quadrata*, disturbance levels of beaches are pristine (black), moderately impacted by humans (red), heavily impacted by humans (blue), heavily impacted by humans and vehicles (green).

As expected, we found that stomach volume increased with dry body mass for each of the species examined. This relationship was, however, highly variable across species. The relationship for some species was approximately isometric, while for others the relationship was positively allometric (slope > 1.0) or negatively allometric (slope < 1.0). We also found that the relationship differed between males and females for some species; however, it did not for others (Table 1, Fig 1). Habitat also had an important impact, as the relationship between stomach volume and dry body mass differed across habitats for both *A*. *pisonii* and for *O*. *quadrata* (Table 1, Fig 2).

**Fig 3:**
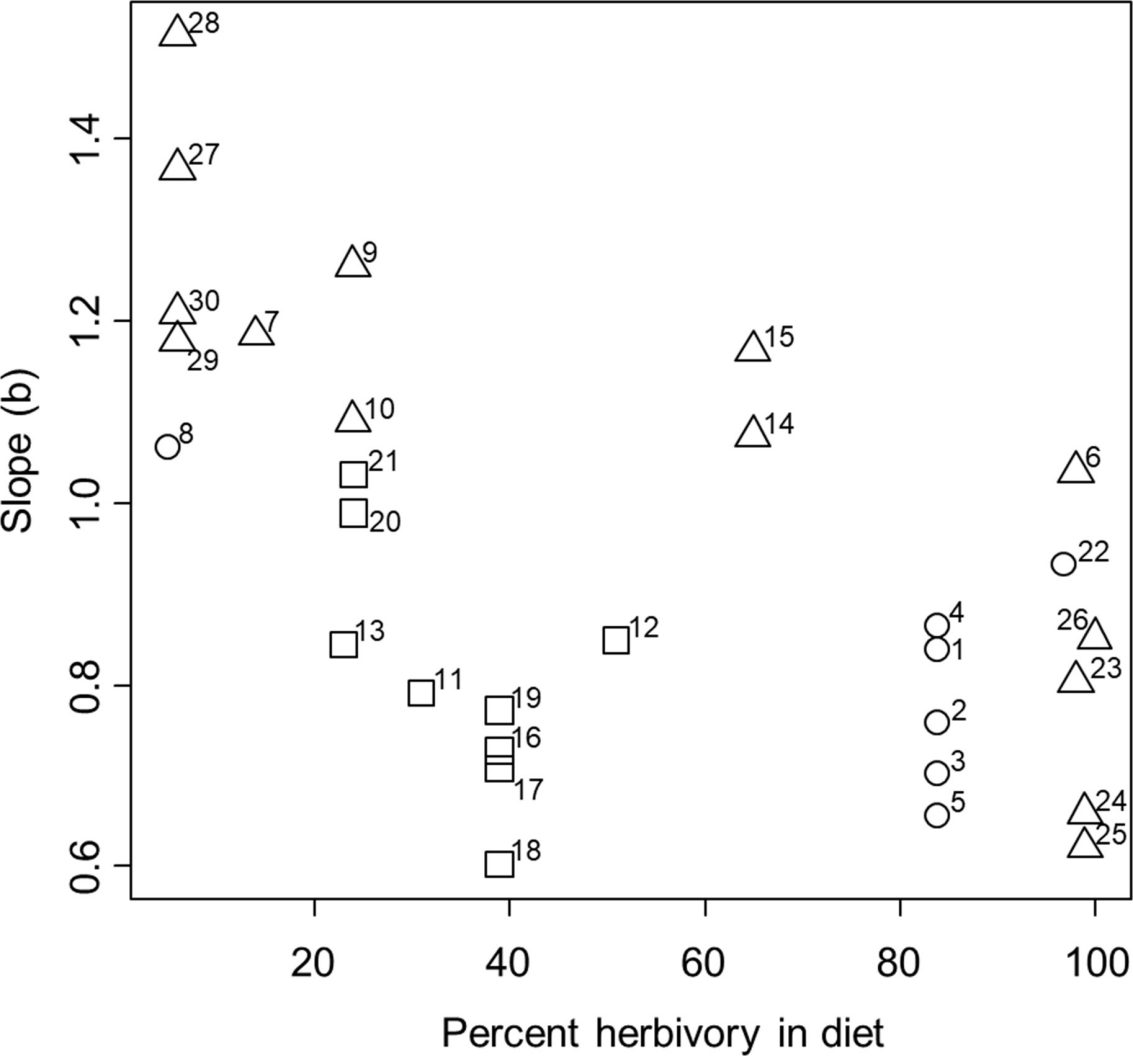
Relationship between the slope of the ontogenetic allometric relationship between log stomach volume and log dry body mass and several ecological factors. Percent herbivory in the diet is shown on the x-axis. Activity levels of crabs were binned for graphical presentation and are shown by the symbol shape, with squares indicating species that are relatively inactive, circles indicating species that are moderately active, and triangles indicating species that are highly active. Numbers next to symbols indicate the species/gender/habitat as follows: 1 –*A*. *pisonii* mangrove male; 2 –*A*. *pisonii* mangrove female; 3 –*A*. *pisonii* marsh male; 4 –*A*. *pisonii* marsh female; 5 –*A*. *pisonii* dock; 6 –*A*. *cinereum*; 7 –*C*. *sapidus*; 8 –*C*. *irroratus*; 9 –*C*. *maenas* male; 10 –*C*. *maenas* female; 11 –*D*. *sayi*; 12 –*E*. *depressus*; 13 –*E*. *limosum*; 14 –*H*. *sanguineus* male; 15 –*H*. *sanguineus* female; 16 –*P*. *herbstii* male; 17 –*P*. *herbstii* female; 18 –*P*. *obesus* male; 19 –*P*. *obesus* female; 20 –*R*. *harrisii* male; 21 –*R*. *harrisii* female; 22 –*S*. *reticulatum*; 23 –*U*. *minax*; 24 –*U*. *pugilator* male; 25 –*U*. *pugilator* female; 26 –*U*. *pugnax*; 27 –*O*. *quadrata* pristine; 28 –*O*. *quadrata* medium human impact; 29 –*O*. *quadrata* high human impact; 30 –*O*. *quadrata* high human impact + vehicles.

**Fig 4:**
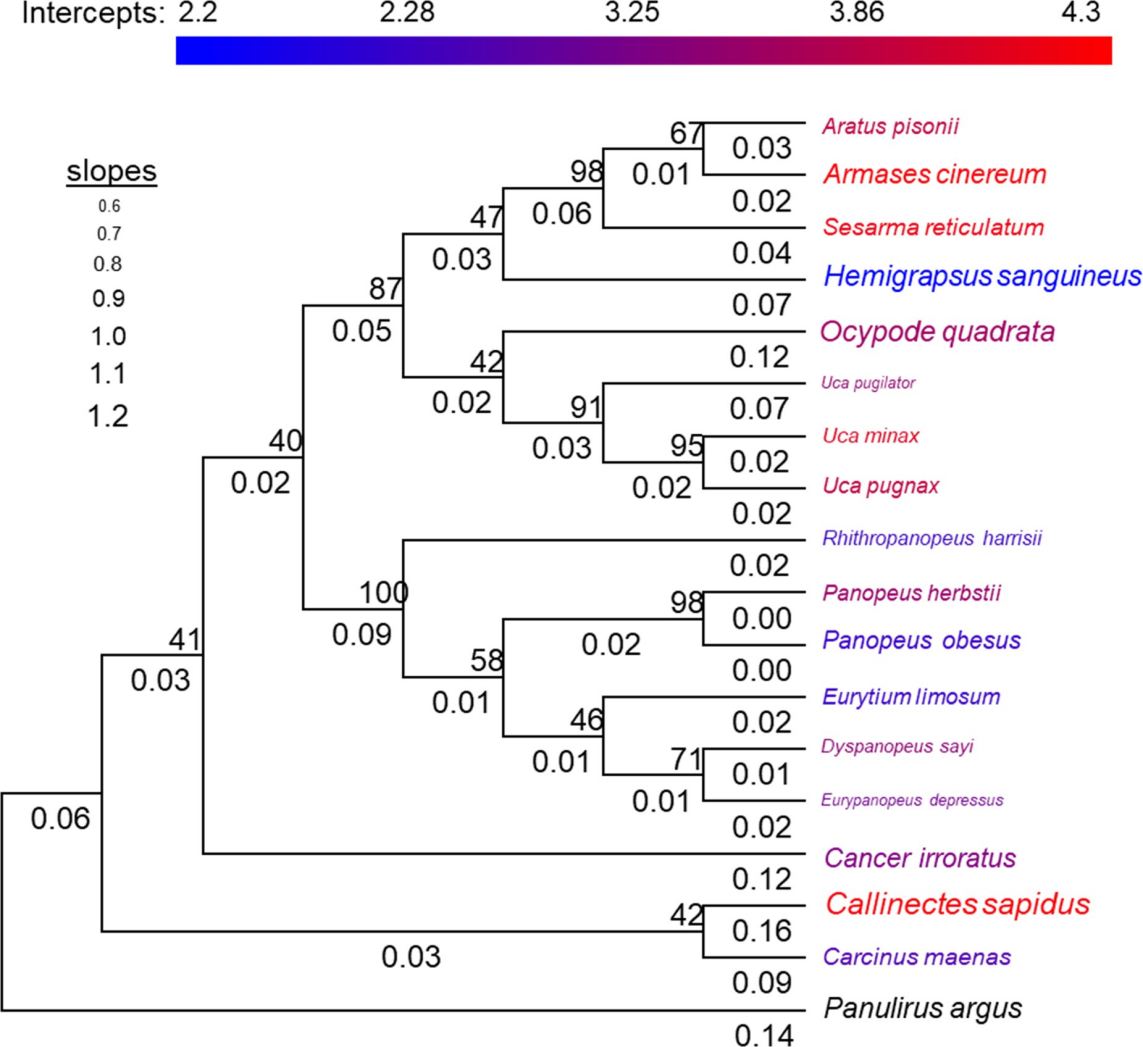
Phylogenetic relationship between crabs used in this study based on overlapping regions of 16S rRNA. The size of the text for the species name gives the slope of the allometric relationship for stomach volume vs. dry body mass (see reference values given on left-hand side of figure for numerical values of different relative sizes), while the color of the text for the species name gives the value of the intercept based on the scale bar shown above the phylogenetic tree. Decimal values given below each branch indicate the branch length, while the whole numbers next to each node indicate bootstrap percentages based on 1,000 bootstrap replicates.

The results showed mixed support for our second hypothesis. Specifically, after controlling for phylogenetic relationship within the mixed-effects model, we found that the slope of the allometric relationship was negatively correlated with percent herbivory in the diet across species, which was opposite of our hypothesis, though the influence was fairly weak (parameter estimate: -0.005 ± 0.001, *t* = -4.98, *P* = 0.0006, Fig 3). Also contrary to our hypothesis, we found that slope was not influenced by the maximum size reached by the species within our samples (*t* = -1.16, *P* = 0.26). However, consistent with our hypothesis, we found that the slope was strongly and positively correlated with activity level across species (parameter estimate: 0.177 ± 0.033, *t* = 5.41, *P* = 0.0004, Fig 3). These trends were weakly influenced by phylogenetic relationship at the genus level (variance explained at the random effect of genus level = 0.003 ± 0.058, with no additional variation explained by the family or species levels, and residual variance = 0.011 ± 0.104, Fig 4).

**Fig 5:**
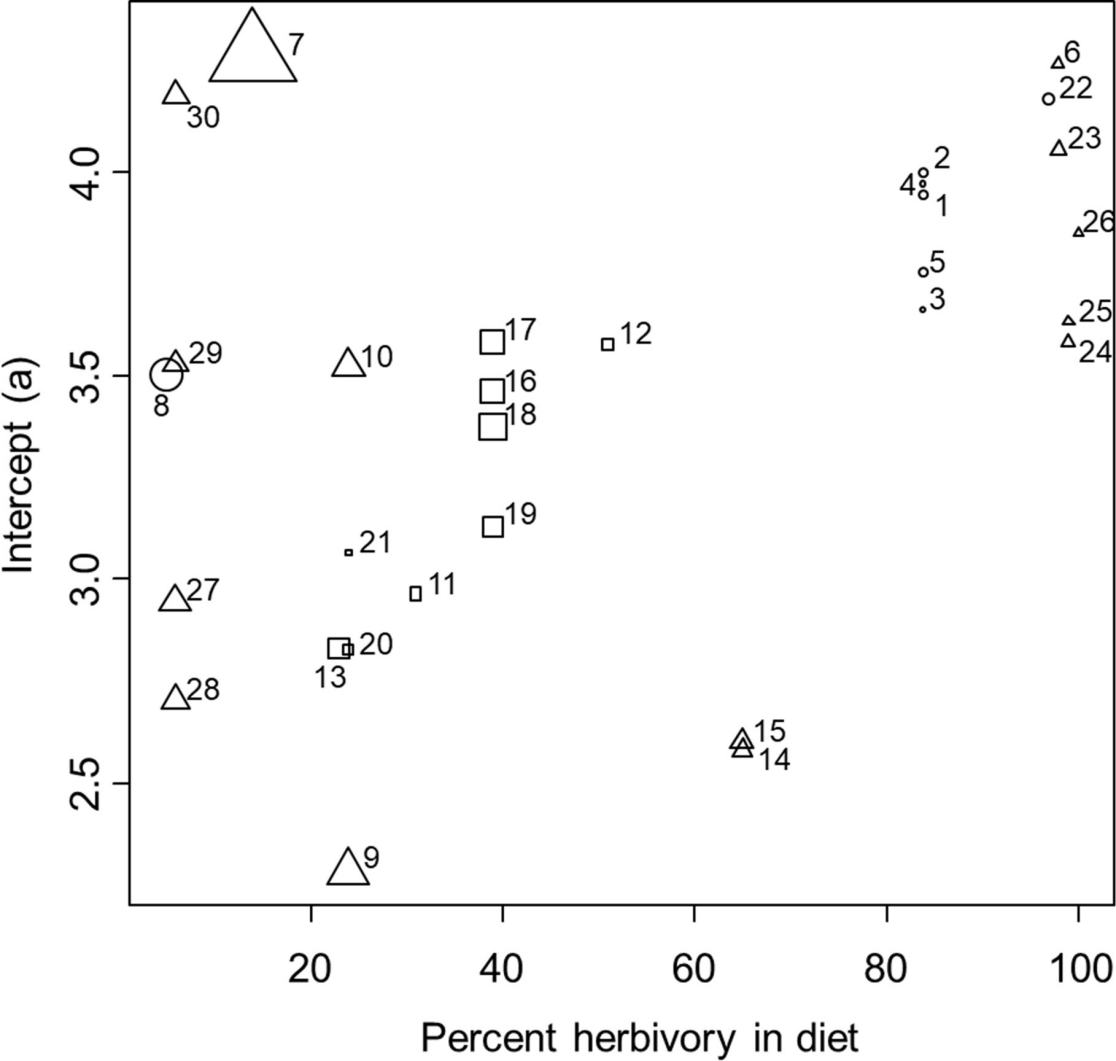
Relationship between the intercept of the ontogenetic allometric relationship between log stomach volume and log dry body mass and multiple ecological factors. Percent herbivory in the diet is shown on the x-axis. Symbol size indicates the relative maximum size of the group or species. Symbol shapes and numbering are as described in Fig 3.

Results for the intercept analysis also showed mixed support for our third hypothesis. Consistent with our hypothesis, and after controlling for phylogenetic relationship within the mixed-effects model, we found that the intercept of the allometric relationship was weakly, but positively correlated with percent herbivory in the diet (parameter estimate: 0.009 ± 0.003, *t* = 2.90, *P* = 0.004, Fig 5) and also increased strongly with the maximum size reached by species within our samples (parameter estimate: 1.414 ± 0.595, *t* = 2.37, *P* = 0.018, Fig 4). However, contrary to our hypothesis, we found no evidence that the intercept was influenced by the activity level across species (activity not included in the best fitting model based on AIC). These trends were strongly influenced by phylogenetic relationship at the family level (variance explained at the random effect of family level = 0.118 ± 0.334, with no additional variation explained by the genus or species levels, and residual variance = 0.147 ± 0.384, Fig 4). Results were similar when *L* was used as an estimate of the elevation of the allometric relationship to insure independence from the slope. Specifically, *L* increased weakly with percent herbivory in the diet (parameter estimate: 0.21 ± 0.03, *t* = 6.25, *P* < 0.0001), it increased strongly with maximum body size reached by species within our samples (parameter estimate: 11.9 ± 4.57, *t* = 2.60, *P* = 0.014), and it was not influenced by activity level across species.

## Discussion

We have shown that stomach volume in brachyuran crabs scales with body size, with a slope that varies from 0.60 ± 0.03 to 1.52 ± 0.13, depending on the species, and that this varies between sexes for some species, but not for others. It is therefore clear that while the stomachs of some species examined here grow isometrically with body mass, consistent with previous assumptions [60], for many other species, the stomach volume increases either faster (slope > 1.0) or slower (slope < 1.0) than isometrically with mass gain. We have also shown that the steepness of the slope was influenced by ecological factors, specifically that it decreased weakly with the amount of herbivory in the diet and increased strongly with activity level. Similarly, we have shown that the intercept of the allometric relationship also varies across and within species and is also influenced by ecological factors; specifically, the intercept increased weakly with the amount of herbivory in the diet and strongly with the maximum size of the species. Likely because of differences in ecological factors across habitats, we also found that the allometric relationships differed across habitats for both of the species where habitat was examined. These results have several implications for brachyuran crabs and potentially for other ectotherms.

Our results demonstrate the importance of diet for shaping the size of the crab stomach, both in terms of the rate of change in stomach size through ontogeny (slope) and in terms of the initial stomach size in juveniles (intercept). Previous work has highlighted variation in crab stomach size that correlates with diet [35, 61]. Diet not only has a strong influence on stomach morphology, but has a strong influence on other diet-related features of crab morphology as well, including claw morphology [62] and morphology of mouth appendages and the gastric mill [63]. These morphological changes in response to diet appear to be highly plastic, differing across seasons in response to seasonal dietary shifts, and across individuals with different dietary preferences [35].

We found that activity level of a species influenced the slope and maximum body size of a species influenced the intercept of the allometric relationship; however, there were no conditions where these two variables were simultaneously influential. This could possibly suggest a tradeoff between these two characteristics. While there is individual variation in body size resulting from individual experience (foraging success, injury, reproductive success), maximum body size of a crab species is a relatively fixed factor given the combination of longevity and molt schedule. The strong, positive influence of maximum body size on the intercept of the allometric relationship of stomach volume and body mass may reflect an evolved strategy of providing species that have high growth potential with large storage capacity that will enable them to consume the large amount of food required for rapid growth. In contrast, activity level differs across species, but is also highly plastic across individuals (e.g., [64]). The strong, positive influence of activity level on the slope of this allometric relationship suggests that an active lifestyle may stimulate more rapid growth of the cardiac stomach to enable sufficient consumption to support high levels of activity. Higher plasticity in stomach growth (slope) as opposed to initial stomach size (intercept) could potentially also explain the greater influence of phylogenetic relationship in patterns of the intercept from this allometric relationship across species compared to patterns in the slope.

We only examined a small subset of the potentially important ecological drivers of changes in allometric scaling. Witting [21] theoretically demonstrated that the slope of the scaling relationship for numerous biological features with body mass can vary with the spatial dimensionality of foraging (i.e., whether organisms forage in 1, 2, or 3 dimensional space). It was predicted that slopes around 0.75 should be more common when organisms foraged in 2-dimensional space, such as for terrestrial organisms, while higher slopes should be more common when organisms forage in 3-dimensions, such as pelagic organisms. The species examined here include a mix of those that commonly forage in 2-dimensional space and those that forage in 3-dimensional space (in mangrove canopies, within oyster reefs, etc.). Thus, dimensionality of foraging may also have contributed to variation in slopes observed here.

Finally, results here may have implications for the response of crabs, and potentially other ectothermic organisms, to environmental changes, such as climate change, that increase their energy demands. As highlighted above, we found that the slope and intercept of the allometric relationship were strongly associated with activity level and the maximum body size, respectively. Shrinking body sizes [65] and increased activity level are two of the consequences of a warming climate for ectotherms. In general, larger body sizes and lower activity levels were characteristics indicative of the carnivorous species examined here, while herbivores were generally smaller and more active. This suggests that warming conditions associated with climate change could cause carnivorous species to shift more towards the patterns of body size and activity level seen in herbivores. Whether the cardiac stomach is able to adapt to these changes through initial stomach size (intercept) or through changes in stomach size during growth (slope) may have important implications for the ability of carnivores to continue to meet their energetic and nutrient demands across ontogeny. Given the limited space available under the crab carapace, changes in gut size to meet these challenges will necessarily result in tradeoffs in the growth or size of other vital organs that compete for the same space ([66], and references therein).
